# Implementation and effectiveness of non-pharmaceutical interventions, including mask mandates and ventilation, on SARS-CoV-2 transmission (alpha variant) in primary schools in the Netherlands

**DOI:** 10.1371/journal.pone.0305195

**Published:** 2024-06-17

**Authors:** Koen M. F. Gorgels, Suhreta Mujakovic, Eline Stallenberg, Volker H. Hackert, Christian J. P. A. Hoebe

**Affiliations:** 1 Department of Sexual Health, Infectious Diseases and Environmental Health, Living Lab Public Health Mosa, South Limburg Public Health Service, Heerlen, The Netherlands; 2 Department of Social Medicine, Care and Public Health Research Institute (CAPHRI), Maastricht University, Maastricht, The Netherlands; 3 Department of Medical Microbiology, Infectious Diseases and Infection Prevention, Care and Public Health Research Institute (CAPHRI), Maastricht University Medical Centre (MUMC+), Maastricht, The Netherlands; Los Alamos National Laboratory, UNITED STATES

## Abstract

There has been a lot of discussion about the role of schools in the transmission of severe acute respiratory coronavirus 2 (SARS-CoV-2) during the coronavirus 2019 (COVID-19) pandemic, where many countries responded with school closures in 2020. Reopening of primary schools in the Netherlands in February 2021 was sustained by various non-pharmaceutical interventions (NPIs) following national recommendations. Our study attempted to assess the degree of regional implementation and effectiveness of these NPIs in South Limburg, Netherlands. We approached 150 primary schools with a structured questionnaire containing items on the implementation of NPIs, including items on ventilation. Based on our registry of cases, we determined the number of COVID-19 cases linked to each school, classifying cases by their source of transmission. We calculated a crude secondary attack rate by dividing the number of cases of within-school transmission by the total number of children and staff members. Two-sample proportion tests were performed to compare these rates between schools stratified by the presence of a ventilation system and mask mandates for staff members. A total of 69 schools responded. Most implemented NPIs were aimed at students, except for masking mandates, which preferentially targeted teachers over students (63% versus 22%). We observed lower crude secondary attack rates in schools with a ventilation system compared to schools without a ventilation system (1.2% versus 2.8%, p<0.01). Mandatory masking for staff members had no effect on the overall crude secondary attack rate (2.0% versus 2.1%, p = 0.03) but decreased the crude secondary attack rate among staff members (2.3% versus 1.7%, p<0.01). Schools varied in their implementation of NPIs, most of which targeted students. Rates of within-school transmission were higher compared to other studies, possibly due to a lack of proper ventilation. Our research may help improve guidance for primary schools in future outbreaks.

## Introduction

There has been a lot of discussion about the role of schools in the transmission of severe acute respiratory coronavirus 2 (SARS-CoV-2) during the coronavirus 2019 (COVID-19) pandemic. Many countries, including the Netherlands, implemented prolonged school closures as part of their efforts to mitigate transmission. However, school closures affect learning opportunities and the mental wellbeing of children and should therefore be considered a measure of last resort in pandemic response [1].

Perceptions of the role of schools in transmission changed during the pandemic. At the pandemic’s outset, children were regarded to be less infectious than adults, and the risk of secondary transmission of SARS-CoV-2 at primary schools was considered to be low [2–4]. As the alpha variant (B.1.1.7) and its successors delta (B.1.617.2) and omicron (B.1.1.529) gained prominence, numerous reports detailing school outbreaks surfaced [5,6]. These reports highlight transmission occurring among students, prompting the implementation of more restrictive infection control measures within educational institutions. In the Netherlands, primary schools were temporarily closed between December 17, 2020 and February 9, 2021. Outside this time frame, a variety of non-pharmaceutical interventions (NPIs) were recommended to mitigate transmission in schools, including a stay-home advice for symptomatic individuals, physical distancing between teachers and students, staggered school start and finish times, staggered break times, and facial mask mandates for teachers and students grade 7–8 [7,8]. Little is known about the actual implementation of these recommendations. One British study which assessed the feasibility of the implementation of NPIs concluded that various measures, including staggered break times and keeping the same staff assigned to one student group, were difficult to implement [9]. Our study assessed to which extent primary schools implemented NPIs, including measures to improve ventilation.

Assessing the effectiveness of these NPIs is inherently challenging, with various methodologies employed in studies. One common approach involves comparing overall attack rates or secondary attack rates post-exposure [10,11]. Alternatively, studies may focus on the ratio between cases stemming from community transmission and those originating within schools [11–13]. To gauge the effectiveness of NPIs with particular emphasis on mask mandates and ventilation, our study conducted an epidemiological analysis, calculating a crude secondary attack rate based on within-school transmission cases at a time when the alpha variant was predominant. Our analysis integrated questionnaire data, publicly available information, and regional epidemiological data from notified COVID-19 cases in the region of South Limburg.

## Methods

### Setting

Primary schools in the Netherlands host children aged 4–12 (grades 1–8). Children were not eligible for vaccination at the time of the study, whereas vaccination for adults, including teaching staff in schools, had become available just prior to the beginning of our study period which lasted from February 2021 till June 2021, a time when alpha had become the dominant variant of SARS-CoV-2. The region of public health service (PHS) South-Limburg has a population of approximately 600,000 inhabitants and consists of 150 primary schools. School size ranged between 74 and 674 children.

Primary schools were closed between December 9, 2020 and February 9, 2021. Dutch health authorities issued a range of mandatory and recommended measures regarding infection prevention measures [7,8,14]. Schools were required to implement mandatory measures under all circumstances, and they were expected to try to implement all recommended measures to the best of their ability. All children with COVID-19 related symptoms were advised to stay at home and undergo testing. Masking was not mandatory but mask mandates for staff members and children from grade 7–8 were recommended. Social distancing (1.5 meter) was mandatory for teaching staff but not for children. Indoor sports outside of schools was prohibited for children of all ages while outdoor sports were allowed. Staggered school start and ending times and staggered break times were mandatory to minimize contacts between different school classes. Staff meetings or meetings with parents had to be held online. An overview of the NPIs evaluated in this study is provided in the supporting information. On June 28, 2021, most restrictions and mandates imposed by Dutch health authorities were lifted by ministerial order, due to decreased levels of SARS-CoV-2 transmission.

### Quarantine and testing protocols

According to national guidelines pertinent at the time of the study, children and staff exposed in a primary school class setting were required to self-isolate at home (home quarantine) for ten days if a SARS-CoV-2 positive case had attended the classroom from two days before symptom onset [15]. All children and staff from affected classes were asked to have themselves tested twice at a community-based testing site, i.e., once as soon as possible after known in-class exposure, and once five days after exposure. If several classes were affected within the same timeframe, closure of the entire school was to be considered.

### Study design

**Fig 1:**
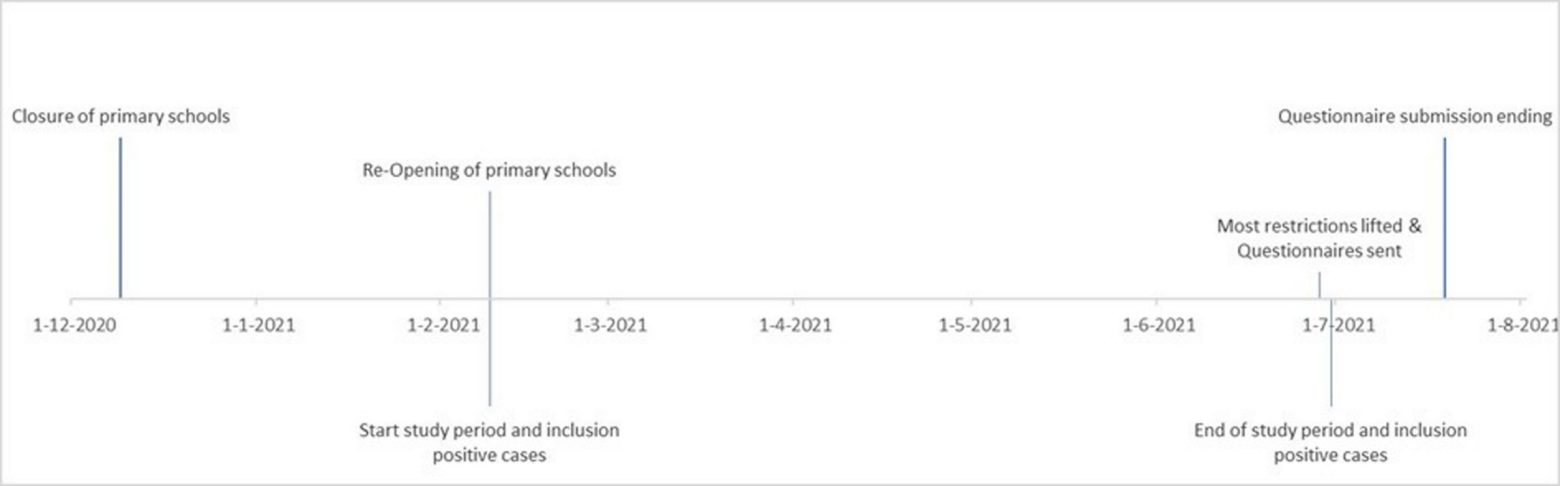
Timeline of study period.

We performed a retrospective study roughly covering a six-month period from February 9 till June 30, 2021. At the end of June 2021, we invited all primary schools in the region of our PHS to participate in a questionnaire survey regarding the implementation of NPIs. The questionnaire covered all NPIs mandated or recommended by the Dutch health authorities from February 9 until June 28, 2021, when schools had reopened after a prolonged period of nationwide school closure. The questionnaire also included questions about ventilation practices and strategies employed by participating schools. Feedback on the questionnaire was provided by schools beforehand. No personal data was gathered in the questionnaire. Failure to respond was followed by a reminder by email and telephone. Fig 1 gives an overview of the study timeline.

### Case definition

Covid-19 cases were individuals who tested positive for SARS-CoV-2 with either a RT-PCR or professionally administered rapid antigen test. All cases had to be reported to the PHS per the Dutch Public Health Act. Testing was provided free of charge to all symptomatic citizens by the regional PHS. Prioritized testing was available for teachers and during suspected outbreak tests were conducted at the location of the school. Cases were followed up through individual source and contact tracing performed by trained PHS professionals, including questions on locations visited during the incubation period and presumable sources. Data gathered from routine source and contact tracing was used in the epidemiological analysis and after initial collection researchers had no access to identifying data. Source and contact tracing was conducted by trained professionals asking cases (or parents/guardians of cases) about their contacts, which locations they had visited and asked about the most likely location in which transmission occurred. All cases linked to a responding school between February 9 and June 30 were included. Cases were further classified as school children or staff members.

### Data analysis

To analyse the data we used SPSS version 26.0 (IBM, Armonk, USA, 2022) and R 4.2.0 (released April, 2022). We compared differences in the implementation of NPIs between the schools. Schools whose response was incomplete with less than five out of 44 questions answered were excluded from analysis as no data on NPIs was given. To assess differences between included and excluded schools, we compared the average size and average number of cases including ranges. School size was determined using publicly available data.

Further analysis was performed on all responding schools. A crude overall attack rate during our study period for children and staff members was calculated using all cases linked to these schools. Cases were further classified based on their probable source: 1.within-school, 2.community transmission and 3.unknown source. **Within-school transmission** was classified as “Cases self-reporting the school as their probable source of transmission OR testing positive after reporting exposure to a known covid case at school while mentioning no other possible source”. **Community transmission** was classified as “Cases reporting an outside source (e.g. a household member or a social visit) as their probable source of transmission”. **Unknown source** was classified as “Cases who reported no probable source or reported both the school and community transmission as possible source”. Due to the unavailability of reliable data regarding the number of exposed individuals for each case, we resorted to calculating a crude secondary attack rate by dividing the total number of children and staff members by the number of cases of within-school transmission. Confidence intervals were computed using the Agresti-Coull method, aligning with previous research [10].

We quantified the implementation of mandatory and recommended measures in each school and assessed their corresponding crude secondary attack rates. As hand sanitizers and collective hand washing fall under the same NPI category, our subsequent analyses focused solely on collective hand washing. To explore the impact of the number of mandatory or recommended measures on within-school transmission rates, we calculated Pearson correlation coefficients. Additionally, we grouped schools based on their implemented measures and compared crude secondary attack rates, as well as rates associated with community and unknown transmission. Two-sample proportion tests were conducted to discern any significant differences between these groups.

An additional analysis was performed to gain a better understanding of the effectiveness of the use of ventilation and face masks as these have been linked to a decrease in cases in the literature [13,16–18]. Our analysis involved comparing the crude secondary attack rates and examining the attack rates associated with community transmission and unknown transmission, considering the number of implemented measures in schools, and stratifying the data based on the presence of a ventilation system. Similarly, we examined the effects of mask mandates for staff members in which we also calculated the crude secondary attack rate among staff members. We did not separately analyze mask mandates for children, as the requirement to wear masks only applied to children in grades 7–8.

### Ethical statement

In the Netherlands, research is required to undergo review by an accredited Medical Research Ethics Committee if it is subject to the Dutch Medical Research Involving Human Subjects Act (WMO). Retrospective research (that is carried out on existing patient material and/or existing patient files) is exempt from the WMO, according to the Dutch Central- Committee on Research Involving Human Subjects (CCMO). Data gathered in the questionnaire was collected during routine infectious diseases activities and did not include any individual data. Data on cases linked to schools were retrospectively retrieved from regular infectious disease control activities and were de-identified. Only anonymized data was used in our analysis and therefore no consent was required. As such, our study does not fall under the scope of the WMO and therefore is exempt from medical ethical approval. This was confirmed by the Maastricht University Medical Centre Medical Ethics Committee (METC 2021–2901).No additional administrative permissions were required to use the data as it is owned by the South Limburg Public Health Service.

## Results

### Study population

The questionnaire was sent to 150 primary schools. Results of 69 schools were included in the analysis of which 59 returned a fully completed questionnaire (total response 46%). The average school size was 248 (range 74–647) for the responding schools and 228 (range 78–551) for the excluded schools. The average number of cases per school was 13 (range 0–59) in included and 14 (range 0–60) in excluded schools.

### Implementation of NPIs

Out of 69 included schools, nine were temporarily closed due to a COVID-19 outbreak, two due to staffing shortages (mostly COVID-19 related, with teachers absent due to isolation or quarantine). Around 20% of all teachers taught several classes.

Implementation of NPIs is shown below in Table 1. Self-reported implementation of NPIs aimed at children was high with most respondents implementing hand washing at fixed moments during the day (86%), different break times between classes (97%) and not allowing parents into the building (100%). Implementation of NPIs for staff members appears more challenging with only 68% switching to online team meetings. Facial mask mandates were higher for staff members (63%) than for students grade 7/8 (22%).

**Table 1:** An overview of implemented measures by primary schools.

Recommended and optional measures	Implemented the measure n/N (%)
**NPIs aimed at children/parents**	
*Mandatory measures*	
Limited access to school building for parents	62/62 (100%)
Staggered break times	60/62 (97%)
Installation of hand sanitizer dispensers	56/59 (95%)
Collective hand washing at fixed moments during the day	51/59 (86%)
Staggered lesson times	26/62 (42%)
*Recommended measures*	
No across-classes lessons	61/69 (88%)
Fixed walking routes	54/62 (87%)
No mixing of classes during breaks	52/62 (84%)
Facial masks recommended for students grade 7–8	13/59 (22%)
*Other measures implemented by schools*	
Closed cafeteria or dining hall	56/59 (95%)
Fixed seating arrangement for students every day	57/63 (90%)
Suspension of visitor access for volunteers	50/63 (79%)
Cohorting of students in groups of up to 6 students	34/62 (55%)
Grouping of students in pairs (2 students)	8/62 (13%)
Maintaining 1.5 meter physical distance between students	4/62 (6%)
**NPIs aimed at teachers**	
*Mandatory measures*	
Team meetings online	40/59 (68%)
*Recommended measures*	
Facial masks recommended for teachers outside the classroom	37/59 (63%)
*Other measures implemented by schools*	
Limited number of attendees during staff meetings	49/59 (83%)
Staff room closure	40/59 (68%)

In the open questions regarding perceived barriers and lessons learned, two respondents mentioned a lack of compliance regarding general infection prevention measures including physical distancing by parents or children outside of school.

### Ventilation conditions and measures

In total, 57% (35/61) of respondents had a working ventilation system present in the whole building, whereas 26% (16/61) had no ventilation system (Table 2). All but two schools took additional ventilation measures, including permanent opening of windows and doors, or repeated temporary opening during breaks. Only two schools reported the use of fans or high-efficiency particulate air filters.

**Table 2:** An overview of ventilation conditions and measures according to primary schools.

Ventilation system	n/N (%)
Working ventilation system in the whole building	35/61 (57%)
Working ventilation system in parts of the building	10/61 (16%)
No	16/61 (26%)
**Ventilation measures taken**	
Windows and doors open during breaks*	42/59 (71%)
Windows and doors constantly open	40/59 (68%)
Use of fans	2/59 (3%)
Use of high-efficiency particulate air filters	2/59 (3%)
No ventilation measures taken	2/59 (3%)

* = Mandatory measure.

### Analysis of individual cases

During the study period, a total of 760 cases in children and 134 cases in staff members were linked to the 69 responding schools. The overall attack rate among all cases regardless of their source of infection was 4.8% (894/18616, 95% CI 4.5–5.1%), 4.4% (760/17112, 95% CI 4.1–4.8%)) among children and 8.9% (134/1514, 95% CI 7.5–10.4%) among staff members. Of these 894 cases, 354 (39.6%) were classified as within-school transmission, 417 (46.6%) cases as community transmission and 123 (13.8%) as unknown source (Table 3). The overall crude secondary attack rate was 1.9% (354/18616, 95% CI 1.7–2.1%), 1.8% (299/17112, 95% CI 1.6–2.0%) for children and 3.6% (55134/1514, 95% CI 2.8–4.7%) for staff members.

**Table 3:** Overview of cases linked to the schools.

	Cases/total N	Overall attack rate	Within-school transmission	Source unknown	Community transmission
Total	894/18616	4.8% (95% CI 4.5–5.1%)	354	123	417
Children	760/17112	4.4% (95% CI 4.1–4.8%)	299	75	386
Staff cases	134/1514*	8.9% (95% CI 7.5–10.4%)	55	48	31

* = Based on self-reported total number of staff members.

### Overall effectiveness of implementation of NPIs

On average, schools implemented 8.4 Fmandatory or recommended measures (Table 4). The number of implemented mandatory or recommended measures were not correlated with crude within-school transmission rates (r = 0.04, p = 0.78). We subsequently categorized schools into two roughly equal groups (Table 4). Results of the two-sample proportion test indicated that there is a significant difference between schools that implemented eight or less measures (1.7% 95% CI 1.4–2.0%) and schools that implemented nine or more measures (2.3% 95% CI 2.0–2.6%) (p<0.01).

**Table 4:** Overview of attack rates schools stratified by the number of implemented measures.

	Average school size	Attack rate community transmission	Attack rate unknown transmission	Crude attack rate within-school transmission
Schools with eight or less implemented measures (n = 26)	261	2.3% (153/6776) 95% CI 1.9–2.6%	0.9% (58/6776) 95% CI 0.7–1.1%	1.7% (112/6776) 95% CI 1.4–2.0%
Schools with nine or more implemented measures (n = 33)	260	2.3% (198/8592) 95% CI 2.0–2.6%	0.6% (55/8592) 95% CI 0.5–0.8%	2.3% (200/8592) 95% CI 2.0–2.6%

CI = Confidence interval.

### Effectiveness of ventilation

Our analysis revealed (Table 5) that the crude attack of within-school transmission rate is lower in schools with a ventilation system in the entire building (1.2% 95% CI 1.0%-1.5%) when compared with schools with no ventilation system (2.8% CI 2.3%-3.3%) (p<0.01) with no difference in the number of infection prevention measures implemented per school and similar attack rates due to community transmission and unknown transmission.

**Table 5:** Overview of attack rates schools stratified by ventilation system.

	Average number of mandatory or recommended measures implemented	Attack rate community transmission	Attack rate unknown transmission	Crude attack rate within-school transmission
Schools with working ventilation system in the whole building (n = 35)	8.4*	2.4% (218/9225) 95% CI 2.1%-2.7%	0.6% (57/9225) 95% CI 0.5%-0.8%	1.2% (112/9225) 95% CI 1.0%-1.5%
Schools with working ventilation system in parts of the building (n = 10)	8.1	2.6% (57/2184) 95% CI 2.0%-3.4%	0.8% (17/2184) 95% CI 0.5%-1.3%	3.3% (72/2184) 95% CI 2.6%-4.1%
Schools with no ventilation system (n = 16)	8.5	2.0% (92/4681) 95% CI 1.6%-2.4%	0.8% (36/4681) 95% CI 0.6%-1.1%	2.8% (130/4681) 95% CI 2.3%-3.3%

*Two schools did not complete the entire questionnaire and were excluded from this average.

CI = Confidence interval.

### Effectiveness of mask mandates for staff members

Our analysis revealed (Table 6) no difference in the crude secondary attack rate is in schools with mask mandates for staff members (2.0% 95% CI 1.7–2.3%) when compared to schools without mask mandates for staff members (2.1% 95% CI 1.8%-2.5%) (p = 0.48) with no difference in the number of infection prevention measures implemented per school and similar attack rates due to community transmission and unknown transmission. Our analysis on staff members revealed significantly lower transmission rates in schools with mask mandates (3.1% 95% CI 2.1%-4.6%) when compared to schools without mask mandates (5.6% 95% CI 3.8%-8.1%) (p = 0.03).

**Table 6:** Overview of attack rates schools stratified by mask mandates for staff members.

	Average number of mandatory or recommended measures Implemented*	Attack rate community transmission	Attack rate unknown transmission	Crude attack rate within-school transmission in students and staff members	Crude attack rate within-school transmission staff members
Schools with facial mask mandate for staff members (37)	7.7	2.2% (202/9162) 95% CI 1.9%-2.5%	0.7% (62/9162) 95% CI 0.5%-0.9%	2.0% (180/9162) 95% CI 1.7–2.3%	3.1% (24/773) 95% CI 2.1%-4.6%
Schools without facial mask mandate for staff members (22)	7.8	2.4% (149/6206) 95% CI 2.0%-2.8%	0.7% (45/6206) 95% CI 0.5%-0.9%	2.1% (132/6206) 95% CI 1.8%-2.5%	5.6% (26/462) 95% CI 3.8%-8.1%

*Not including facial mask mandates for staff members.

CI = Confidence interval.

## Discussion

Our results show that implementation of NPIs varied widely between primary schools, and that uptake was higher for NPIs targeted at children than teaching staff. The overall attack rate among all cases regardless of their source of infection was 4.8% with higher rates among staff members compared to children. Further analysis established that the presence of a ventilation system was significantly associated with reduced rates of within-school transmission. Additionally, schools with mask mandates for staff members had reduced rates of within-school transmission among staff members.

Primary schools varied in their implementation of NPIs. Most schools implemented at least some measures regarding hygiene and social distancing. The only NPI that was universal across all participating schools was barring parents from entering the school premises. Only 42% of schools implemented staggered lesson times. Interestingly, some schools implemented measures exceeding official mandates or recommendations, including distancing of 1.5 meter between students. Our findings are in agreement with a study from the United Kingdom that revealed a grand majority of schools implemented recommended measures, with some schools reporting difficulties implementing measures such as staggering break and lesson times [9]. Moreover, a U.S. study observed significant variation in the implementation of NPIs in universities [19].

Prevalence of measures targeting teaching staff was comparatively low. Only 68% (40/59) of schools reported switching to 100% online team meetings. Whereas we had no data on the prevalence of hybrid or alternating online versus physical meetings, a study performed in the UK reported 93% of schools implementing a full switch to online meetings [9]. Only 63% of schools asked staff member to wear facial masks, and 20% of teachers taught across several classes. All these factors may have contributed to a higher overall attack rate among teachers compared to children in our study. Higher prevalence of infection in teaching staff was also observed in other European research [10,20,21].

While it is encouraging that most schools implemented measures related to hygiene and social distancing, there is a need for more guidance and support to ensure consistent and effective implementation of NPIs. The fact that some schools went beyond official mandates or recommendations indicates a proactive approach, but it also highlights the lack of uniformity in implementation across different educational institutions. A collaborative effort between education authorities, health organizations, researchers and school communities is crucial to navigate the challenges of future outbreaks.

Our overall attack rate is higher than a German study reported during the alpha variant although the time period observed in that study was only 15 weeks versus our 22 weeks. The number of cases of within-school transmission and subsequent crude secondary attack rate was also higher than reported in other studies [10–13]. Furthermore, the ratio between community transmission and within-school transmission was lower than in other US studies that describe ratios of 12:1, 16:1 and 20:1 [11–13]. Data in these studies were adjudicated by local health departments in partnership with school staff and may have underestimated the number of events of within-school transmission. In contrast, our data on the most probable source of infection were based on reporting by parents, who may be more likely to mention the school as the most probable source of infection, especially when the school had reported cases. Additionally, transmission events with children from the same class occurring outside of school may have erroneously been classified as within-school transmission. Other studies also rapport higher rates of suspected within-school transmission. A study from schools in Norway determined that in 45% of cases of community transmission in schools led to at least one subsequent case within 14 days [22]. Comparing findings across countries is challenging due to variations in methodology, including local testing strategies and the classification of within-school transmission, which limits the generalizability of our results.

Inadequate ventilation may have facilitated within-school transmission. We found that over 25% of schools had no working ventilation system. We calculated lower crude secondary attack rates in schools with a ventilation system in the entire building (1.2% versus 2.8%, p<0.01), without large differences in the number of cases of community transmission or unknown transmission. One caveat in interpreting our results is that the definition of the term ventilation system was not specified in our questionnaire. However, our results align with those of a New York study and an Italian study which found that transmission was increased in classrooms without a mechanical ventilation system, and those of a Dutch report estimating that 27.9% of all primary school buildings did not meet ventilation standards under state legislation, mostly due to lack of a mechanical ventilation system [17,23,24]. Almost all schools included in our study implemented cross ventilation by opening windows and doors, which likely contributed to reduction of transmission. Awaiting structural improvements, optimizing natural ventilation may be a good interim solution.

Determining the precise impact of mask mandates on within-school transmission based on our study is challenging. We found no evidence of reduced transmission in schools with mask mandates for staff members but masks were (usually) not required for children grade 7–8 and never for children grade 1–6 complicating a comprehensive assessment. When limiting our analysis to staff members we saw reduced rates of within-school transmission in schools with mask mandates (3.1% versus 5.6%, p = 0.03) in agreement with earlier literature. Universal masking of both children and staff members has been associated with a decrease in secondary cases and school outbreaks in multiple studies across different time periods and different prevailing variants of SARS CoV-2 and evidence of their effectiveness also exists for other respiratory viruses [10,16,18,25–29]. One important thing to note when interpreting our results is that mandates did not specify whether medical or non-medical masks should be used, which could have influenced their effectiveness in reducing transmission.

Our results suggested that schools which implemented more measures than the sample average had higher within-transmission than school which implemented less measures than the sample average. We speculate that schools with higher within-school transmission subsequently implemented more measures, however, our data did not permit to study variation in the timing of NPIs. Additionally we did not control for ventilation status which could have affected our results.

In contrast, a modeling study suggests a dose-response effect, with each additional layer of infection prevention measures associated with a 7% decrease in COVID-19 incidence [30]. Additionally, a comprehensive German empirical study supports the effectiveness of reduced classroom sizes [10]. Future research should prioritize further investigating the real-world feasibility and effectiveness of NPIs to guide informed policy development.

Our study has several strengths and limitations. A large analysis using multiple data sources gave a clear overview of the implementation of NPIs and some empirical evidence on their effectiveness. Nevertheless, we cannot exclude bias in our results, as the response rate did not exceed 46%.

While relying on individual-reported data for presumed sources of infection and known exposures may introduce the possibility of misclassification bias, it is important to emphasize that these data were collected through telephonic interviews conducted by trained professionals. To minimize investigator bias, we established clear definitions for within-school transmission and community transmission. Nonetheless, it is important to acknowledge that some degree of misclassification bias may still exist. However, we believe this bias to be non-differential, meaning it is unlikely to significantly impact our main conclusions, although it may have influenced the overall results to some extent.

Finally, it should be noted that we lack data regarding individuals’ adherence to the diverse NPI measures implemented by schools, compliance with COVID-19 guidelines outside of school, and there might be variations in the timing of implementation across schools. Multiple respondents mentioned low adherence by parents to physical distancing mandates outside of school, and this may have influenced transmission rates.

Our study findings underscore the significant variation in the implementation of NPIs among primary schools and emphasize the need for additional guidance and support in their implementation. We observed high rates of within-school transmission, which may be partly attributed to a lack of ventilation systems in a number of schools. To effectively address future outbreaks, it is imperative to foster collaboration between researchers, education authorities, health organizations, and school communities. By working together, comprehensive strategies that prioritize the safety and well-being of students and staff members in educational settings can be developed and implemented.

## Supporting information

S1 File Overview of all evaluated measures in this paper. (PDF)

S2 File Questionnaire (English translation). (PDF)

S3 File STROBE-checklist. (DOCX)

S4 File Minimal dataset. (XLSX)
