# Supplementary material for: Implementation and effectiveness of non-pharmaceutical interventions, including mask mandates and ventilation, on SARS-CoV-2 transmission (alpha variant) in primary schools in the Netherlands
Source: PLoS One. 2024 Jun 17;19(6):e0305195. doi: 10.1371/journal.pone.0305195 (PMC11182535; doi:10.1371/journal.pone.0305195)
Supplement: S1 File — (PDF) [file pone.0305195.s001.pdf]

**Overview of all evaluated measures in this paper**

**Mandatory measures**
Measures based on the advice in the generic framework established by the RIVM or set by law. Schools must comply with these measures under all circumstances

- Limit access to school building for parents. Only allow parents inside when staying outside is not possible due to the age of the child in special education. Make sure 1.5 meter distance can be maintained by parents in schools.
- Staggered break times to reduce mixing of classes during breaks
- Staggered lesson times to reduce mixing of classes during the start of school/end of school
- Perform collective hand washing at fixed moments including when entering the classroom. Make sure enough cleaning materials are available for this.
- All staff meetings are to be held online
- Open windows and doors during breaks to increase ventilation

**Recommended measures**
Measures that are not mandatory and may not be feasible in all situations. We expect schools to implement these measures as well as possible, taking into account their own context.

- No lessons in which students are mixed with students from other classes.
- Make sure fixed walking routes are implemented throughout the school.
- No mixing of classes during breaks
- Facial masks recommendation for students grade 7-8. The face mask can be removed when sitting down in the classroom.
- Facial masks recommendation for staff members when teaching grade 7-8 or when teaching younger classes. No specific recommendations are made whether this includes sitting down inside the class.
- Consider cohorting students into groups of 5 for grades 3-8 and consider cohorting students in smaller groups than 5 for grades 7-8*

*As this measure was listed as a consideration we did not include cohorting in either the mandatory or recommended measures.
