# Supplementary material for: Implementation and effectiveness of non-pharmaceutical interventions, including mask mandates and ventilation, on SARS-CoV-2 transmission (alpha variant) in primary schools in the Netherlands
Source: PLoS One. 2024 Jun 17;19(6):e0305195. doi: 10.1371/journal.pone.0305195 (PMC11182535; doi:10.1371/journal.pone.0305195)
Supplement: S2 File — (PDF) [file pone.0305195.s002.pdf]

Questionnaire (English translation)

1 What is the name of your school?

Open answer

2 How many individual groups does your elementary school consist of?

(for example, counting two groups of grade 1 as 2. A combination class of grade 1 and 2 counts as 1 group)

Open answer

3 How many staff members do you have working on location?

Open answer

4 How many staff members at your school have direct contact with children?

Only count people who are in front of the class, including interns and teaching assistants.

Open answer

5 How many teachers/interns/teaching assistants teach multiple groups?

Open answer

6 Were there any lessons held in classrooms where students from different groups were sitting together? This includes, for example, joint music lessons. Do not include gym class because this class is taught in a separate room. Single-response question

Yes

No

7 How large is the average lower-level group? Single-response question

30+

26-30

21-25

20

8 How large is the average upper-level group? Single-response question

More than 30

26-30

21-25

20 or less

9 Was the school closed? Single-response question

Yes, due to an outbreak

Yes, due to a shortage of teachers

Yes, (other reason)

No

10 Does the elementary school have an after-school care (BSO) or daycare center (KDV) that was open? This concerns a BSO or KDV adjacent to the school building. Single-response question

No

Yes, only BSO

Yes, only KDV

Yes, both

11 Are the supervisors/employees internal or external? (External = not employed by the school; Internal = employed by the elementary school) Single-response question

Yes

No

I don’t know

12 Have the following lessons taken place during the period when schools were open until June 1st?

Single-response question*

Gym with multiple classes together outside

Gym in 1 class outside

Gym with multiple classes inside

Gym with 1 class inside

Drawing/crafts lesson

Music lesson

13 Was there at any time a joint celebration with multiple groups physically present?

Multi-response question

Yes, for carnival

Yes, around King's Day

Yes, around the musical

Yes (other)

No

14 Were parents also allowed to attend?

Multi-response question

Yes, for carnival

Yes, around King's Day

Yes, around the musical

Yes (other)

No

15 Were parents used as volunteers for other activities where they had to be physically present (such as lunchtime supervisors)?

Single-response question

Yes

No

16 Does each student have a fixed seat?

Single-response question

Yes

No

17 Are the students sitting one and a half meters apart from each other?

Single-response question

Yes

No

18 Do students work together in pairs or fixed groups?

Single-response question

Yes, in pairs

Yes, in fixed groups of up to 6 students

No

19 Are there separate breaks?

Single-response question

Yes, groups do not mix on the playground

Yes, but groups can mix on the playground

No, everyone has a break at the same time and groups cannot mix

No, everyone has a break at the same time and groups can mix on the playground

20 In what ways has information been provided about infection prevention measures?

Multi-response question

Personal explanation to the children

Letter/email to parents

Posters distributed throughout the school

Videos for children

Webinar/information session for parents

Information session for teachers

Other, namely (open answer)

21 Were there multiple start and end times of the school day?

For example, to limit the number of children arriving/departing from the school at the same time.

Single-response question

Yes

No

22 Were parents allowed inside during drop-off/pick-up?

Single-response question

Yes

No

23 Do you use fixed walking routes?

Single-response question

Yes

No

24 How many buildings does your school consist of?

Open question

25 In how many classrooms did students go directly from outside to inside without having to walk through the hallway?

Single-response question

All

More than three-quarters

Half to three-quarters

Quarter to half

Less than a quarter

26 Does your school have a functioning ventilation system?

Single-response question

Yes, in the entire building

Yes, in more than half of the classrooms/buildings

Yes, in less than half of the classrooms/buildings

No

27 Does your school comply with the requirements for ventilation according to the building regulations? Single-response question

Yes (confirmed by investigation)

No (confirmed by investigation)

Not checked

28 Has your ventilation system been inspected in the last 2 years? Single-response question

Yes

No

29 Have any measures been taken regarding ventilation? Multi-response question

No

Windows and doors open during breaks

Windows and doors open constantly

Use of fans

Use of air purification machines

30 Are there hand hygiene/disinfection points present at the entrance or in the classroom? Single-response question

Yes

No

31 Was this mandatory at the beginning of each lesson? Single-response question

Yes, in all groups

Yes, in some groups

No

32 Was a canteen open? Single-response question

Yes

No

33 Are the students' workstations cleaned daily by the students themselves? Single-response question

Yes

No

34 Have teachers used face shields? Single-response question

Yes

No

35 How were masks for teachers handled outside the classroom? Single-response question

Mandatory

Optional

At their own discretion

36 How were masks for students in grades 7/8 handled outside the classroom?

Single-response question

Mandatory

Optional

At their own discretion

37 Are self-tests being used? Single-response question

Yes, by teachers

Yes, by students

Yes, by both

No

38 Were all meetings between teachers held digitally? Multi-response question

Yes, all of them

Yes, most of them, but some were still physical

No

39 Was a teachers' room used? Single-response question

Yes

No

40 Were there any agreements about how many teachers could be present at the same time? Single-response question

Yes, max 2 teachers

Yes, max 3-4 teachers

No agreements

41 Have there been any changes in your policy from the time schools reopened until now, and if so, from when and what changes?

Open question

42 Have you taken any other measures that are not mentioned here?

Open question

43 Would you like to mention anything else that you believe contributes to the spread? Open question (small)

Open question

44 Would you have done anything differently in hindsight with the knowledge you have now? Open question (small)

Open question

*This was meant to be a multi-response question. Data from this question was not further analyzed.
